# Mr. Granger's Remarks on Mr. Webster

**Published:** 1813-09

**Authors:** Benjamin Granger

**Affiliations:** Burton-upon-Trent


### *To the Editors of the Medical and Physical Journal.*

GENTLEMEN,

THE account which I sent to the Edinburgh Journal respecting the late investigation at Tutbury, having been strangely mistated by Mr. Webster in your last Number, will you permit me to notice some of his errors and misrepresentations.

He maintains that when the watch on the impostor, Ann Moore, broke up, she exhibited "the usual appearances of a person in a dying state." It is now known, however, that she assumed these alarming appearances to avoid, for obvious reasons, being removed from the bed on which she lay. Having gained this point, the appearances of approaching death *suddenly* vanished. She raised herself up, washed herself, and then said to Mr. Bott, who did not leave the room for some time, "You see, Mr. Bott, I'm not much worse!" As I, therefore, just mentioned, in my paper in the Edinburgh Journal, the circumstance of the illness being in a great degree assumed, when she seemed to be in a dying state, Mr. Webster tells your readers that I deny that she was ill at all, and that I look upon the syncope, the change of pulse, and, I presume, the loss of flesh, as having *nothing* to do with the ten days abstinence, but as being altogether *feigned*. I beg, therefore, to be allowed to correct these misrepresentations, and to say that the opinions which Mr. Webster ascribes to me are to be found only in his *misquotations* of my paper.

Having also mentioned that on the tenth day of her abstinence the woman was in a state of syncope, the pulse *indistinct*,* Mr. Webster makes me say that the pulse *was* 140! and exultingly puts this absurd question—" Whether it was possible to feign (I do not like, he says, the word simulate) syncope with a pulse at 140, and indistinct?" Why Mr. Webster has committed such mistakes in representing my opinions, I can no more explain than I can the rude spirit of contradiction which pervades the whole of his animadversions. I remain, Gentlemen,

*Burton-upon-Trent*, Your most obedient Servant,

*August* 10, 1813. BENJAMIN GRANGER.

* The remarkable acceleration and sudden change of the pulse, will, I think, be best accounted for from the irritation of the over-distended bladder. Dr. Currie and M. Delletan have shown that the pulse undergoes surprisingly little change in cases of inanition.

from

